# Development of a log file analysis tool for proton patient QA, system performance tracking, and delivered dose reconstruction

**DOI:** 10.1002/acm2.13972

**Published:** 2023-03-23

**Authors:** Ozgur Ates, Fakhriddin Pirlepesov, Li Zhao, Chia‐ho Hua, Thomas E. Merchant

**Affiliations:** ^1^ St. Jude Children's Research Hospital Memphis Tennessee USA

**Keywords:** Log file‐based proton patient QA, proton dose reconstruction, performance tracking

## Abstract

**Purpose/Objective(s):**

To describe a log file–based patient‐specific quality assurance (QA) method and develop an in‐house tool for system performance tracking and dose reconstruction in pencil‐beam scanning proton therapy that can be used for pre‐treatment plan review.

**Materials/Methods:**

The software extracts beam‐specific information from the treatment delivery log file and automatically compares the monitor units (MU), lateral position, and size of each spot against the intended values in the treatment plan to identify any discrepancies in the beam delivery. The software has been used to analyze 992 patients, 2004 plans, 4865 fields, and more than 32 million proton spots from 2016 to 2021. The composite doses of 10 craniospinal irradiation (CSI) plans were reconstructed based on the delivered spots and compared with the original plans as an offline plan review method.

**Results:**

Over the course of 6 years, the proton delivery system has proved stable in delivering patient QA fields with proton energies of 69.4–221.3 MeV and an MU range of 0.003–1.473 MU per spot. The planned mean and standard deviation (SD) of the energy and spot MU were 114.4 ± 26.4 MeV and 0.010 ± 0.009 MU, respectively. The mean and SD of the differences in MU and position between the delivered and planned spots were 9.56 × 10^−8^ ± 2.0 × 10^−4^ MU and 0.029/−0.007 ± 0.049/0.044 mm on the X/Y‐axis for random differences and 0.005/0.125 ± 0.189/0.175 mm on the X/Y‐axis for systematic differences. The mean and SD of the difference between the commissioning and delivered spot sizes were 0.086/0.089 ± 0.131/0.166 mm on the X/Y‐axis.

**Conclusion:**

A tool has been developed to extract crucial information about the performance of the proton delivery and monitor system and provide a dose reconstruction based on delivered spots for quality improvement. Each patient's plan was verified before treatment to ensure accurate and safe delivery within the delivery tolerance of the machine.

## 1 INTRODUCTION

Log file–based patient‐specific quality assurance (QA) has been used as a complement to or sanity check for the standard 2D detector array measurements in conventional photon therapy,^1^, ^2^, ^3^ in which each multi‐leaf collimator (MLC) control point for every beam angle contributes to the overall 3D dose distribution. In pencil‐beam scanning (PBS) proton therapy, or spot scanning proton therapy (SSPT), each proton spot or beamlet is tuned to carry a specific energy and dose to an appointed range and lateral position. With the advent of the intensity‐modulated proton therapy (IMPT) technique, highly conformal dose distributions can be achieved by using thousands of individual proton beam spots with modulation of the Bragg peaks.^4^

As a standard approach to proton patient QA, absolute dose measurements via an ionization chamber array detector are performed at selected depths for each field during the pre‐treatment patient‐specific QA. Not only is this time consuming, but the gamma analysis does not necessarily reveal local hot and cold regions at the anatomic level. A recent study^5^ revealed a poor correlation between the gamma passing rates of the pre‐treatment patient‐specific QA for proton therapy and the patient plan dose errors in anatomic regions of interest. To address this shortcoming, a few studies^6^, ^7^, ^8^ have incorporated an independent dose calculation along with the treatment log file analysis, which can provide crucial, granular information about each proton beam spot delivered by the machine.

In PBS proton therapy, each proton spot is individually generated by the proton accelerator, with uncertainties being compounded by the physical characteristics of the spot, such as its MU, position, and size.^9^ When the set tolerances are exceeded, the accelerator is designed to interlock or hold off the beam based on its spot position and data from dose monitors until a new set of spots have been generated with acceptable uncertainties. However, the cumulative effects of multiple spot deviations may still affect the quality of treatment plans and render reconstructed dose assessments necessary to ensure delivery accuracy.^10^, ^11^, ^12^

Here, we report the results of a trend analysis of a log file–based patient‐specific QA that was developed in‐house for pediatric PBS proton therapy. Since 2016, the software has been used to analyze 992 pediatric patients, 2004 plans, 4865 fields, and more than 32 million proton beam spots by using 96 discrete proton energies ranging from 69.4 to 221.3 MeV. To our knowledge, this is the first report of dose reconstruction for pediatric patients receiving craniospinal irradiation (CSI) based on a comparison of spot information recorded in the beam delivery log file against the original treatment plans. We also assess the accuracy of the proton beam delivery to the target and organs at risk (OARs).

## 2 METHODS

This section describes the design and development of the in‐house log file–based proton patient QA tool in sub‐section (2.1), the trend analysis of machine performance and delivery accuracy from 2016 to 2021 over 6 consecutive years in sub‐section (2.2), and the reconstruction of the beam delivery in the treatment planning system (TPS) in sub‐section (2.3).

### 2.1 The design of an in‐house log file–based proton patient QA

Log file–based patient‐specific QA is an integral part of physics QA procedures, especially for SSPT delivery systems, with which a large number of spots form a single radiation field. The in‐house physics software was designed for two purposes: (1) to serve as a sanity check against the vendor's own safety interlock system to verify that all spots created by the machine are delivered within the acceptable specifications and standards in terms of the spot MU, spot position, and spot size; and (2) to check the planned spots from the TPS against the delivered spots, using recorded log files and the MOSAIQ Record and Verify (R&V) system (Elekta Solutions AB, Stockholm, Sweden). The gantry angle of the patient fields for log file–based proton patient QA always matched the gantry angles in the original plan, but 2D absolute dose measurements were performed only at gantry 0° because of the physical limitations of the measuring device. Therefore, the spots delivered at the treatment gantry angle enabled an extra layer of spot verification besides the gamma analysis. Even though the delivered spots were within the machine tolerances, large discrepancies in spot characteristics between planned and delivered spots could still be an issue requiring further evaluation. The software also enables precise determination of the exact spot number and energy layer at the time at which large discrepancies occur.

The machine tolerances corresponding to the *alarm* and *beam abort* levels for spot MU, position, and size have not been disclosed as they are confidential proprietary information specific to the PROBEAT‐V system (Hitachi Ltd., Tokyo, Japan). Our institution implemented the vendor's clinical thresholds in the in‐house software to verify the differences in spot MU and position between the TPS plan and the QA delivery.

MU was defined as the uniform dose of 2.17 Gy delivered to 1 L of water at isocenter using varying energies up to the maximum energy of 174 MeV to form a spread‐out Bragg peak (SOBP) of 10 cm width, 20 cm depth in water with a 10 cm x 10 cm field size, and a total of 217 MU. Minimum and maximum deliverable MU per spot were 0.003 and 5 MU, respectively.

Spot MU checks were based on the MU values per spot from the TPS and were verified against variable alarm and abort levels. Spot position checks on the lateral X‐ and Y‐axes were rather complex and were dependent on random and systematic errors in the positions of the spots and energy layers. The spot MU and position were detected, respectively, by the Hitachi dose monitor and spot position monitors in the nozzle, and they were interpolated into the machine isocenter for TPS comparisons.

**FIGURE 1:**
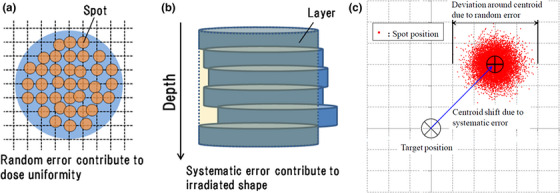
Spot distribution can vary within each energy layer as a result of statistical random error (a), and systematic error causes a shift of the entire layer from its centroid position (b). These errors are the two components of spot position error in the lateral directions X and Y (c).

Figure 1 illustrates how systematic errors affect the positional shifts of the entire energy layer and how random errors can degrade the dose uniformity within the same energy layer. Although systematic error can shift the entire energy layer like a block, random error is responsible for the spot distribution within the energy layer.

Equation 1 formulates the deviation ($D_{spot})$ of the spot position on the X‐axis when the delivered and planned spot positions are compared:

(1) $$D_{spot}=\;X_{delivered}-\;X_{planned}$$

where $X_{delivered}$ is the delivered spot position on the X‐axis and $X_{planned}$ is the planned spot position on the X‐axis.

Equation 2 illustrates the verification of the systematic error against the machine tolerance. This step calculates the sum of the positional differences of each spot divided by the number of accumulated spots by averaging out the shift of the entire cluster or the systematic shift of the layer from the planned position.

(2) $$\frac{\sum_{L\;=\;1}^{N_{spot}}D_{spot}}{N_{spot}}\;\leq Tolerance$$

where $N_{spot}$ is the number of accumulated spots in the layer.

Equation 3 shows the verification of the random error against the tolerance by calculating the individual spot displacement from the centroid position that represents the statistical error of the spots around the centroid.

(3) $$D_{N_{spot}}-\;\frac{\sum_{L\;=\;1}^{N_{spot}}D_{spot}}{N_{spot}}\;\leq Tolerance$$

The spot size, a function of the beam energy, is verified by a spot position monitor for each energy layer. The Hitachi machine has a verified list of values for the combination of gantry angle and energy level obtained during commissioning because the angular position of the steering magnets can affect the size of proton beam spots at different gantry angles. The values detected by the spot position monitor are compared against these clinically accepted values during commissioning.

### 2.2 Trend analysis of the log file–based proton patient QA

A MATLAB‐based algorithm was developed to extract beam‐specific information, including the spot MU, position, and size, from treatment delivery log files recorded by the MOSAIQ R&V system. The software was used to compare the delivered values for the MU and the position on the lateral X‐ and Y‐axes for each spot with the planned values from the TPS to detect any discrepancies in the beam delivery. In the same procedure, the delivered spot sizes were also monitored for various energies and gantry angles as recorded by the spot position monitor and compared with the baseline values established at the time of acceptance and commissioning.

Over the course of 6 years, the in‐house software has been used to verify the treatment accuracy for 992 pediatric patients, 2004 plans, 4865 fields, and more than 32 million proton beam spots as part of the clinical proton patient QA procedure. Institutional review board (IRB) approval was secured to disclose the results of this study.

### 2.3 Reconstruction of the beam delivery in the treatment planning system

The in‐house software can use spot information from the recorded log files and recalculate the original plan in the Eclipse TPS (Varian, Palo Alto, CA) to enable comparisons of intended and delivered dose distributions. Patients undergoing CSI have relatively large target volumes that include disease sites from the cranium through the sacrum. A typical CSI patient in our clinic was treated with two posterior oblique beams for the cranial portion and one or two posterior beam(s) for the spine region, with robust optimization that included setup uncertainties and dose gradient optimization across the junction. CSI patients were prescribed 15 Gy(RBE), 23.4 Gy(RBE), or 36 Gy(RBE) based on the risk categories for the clinical target volumes (CTVs) designated CTV Cranium and CTV Spine. For this study, 10 pediatric patients who received CSI between 2016 and 2020 were randomly selected for reconstruction of the beam delivery in the TPS. The purpose of the study was to detect and pinpoint any local discrepancies in the delivered dose distributions over large areas of patient anatomy.

## 3 RESULTS

**FIGURE 2:**
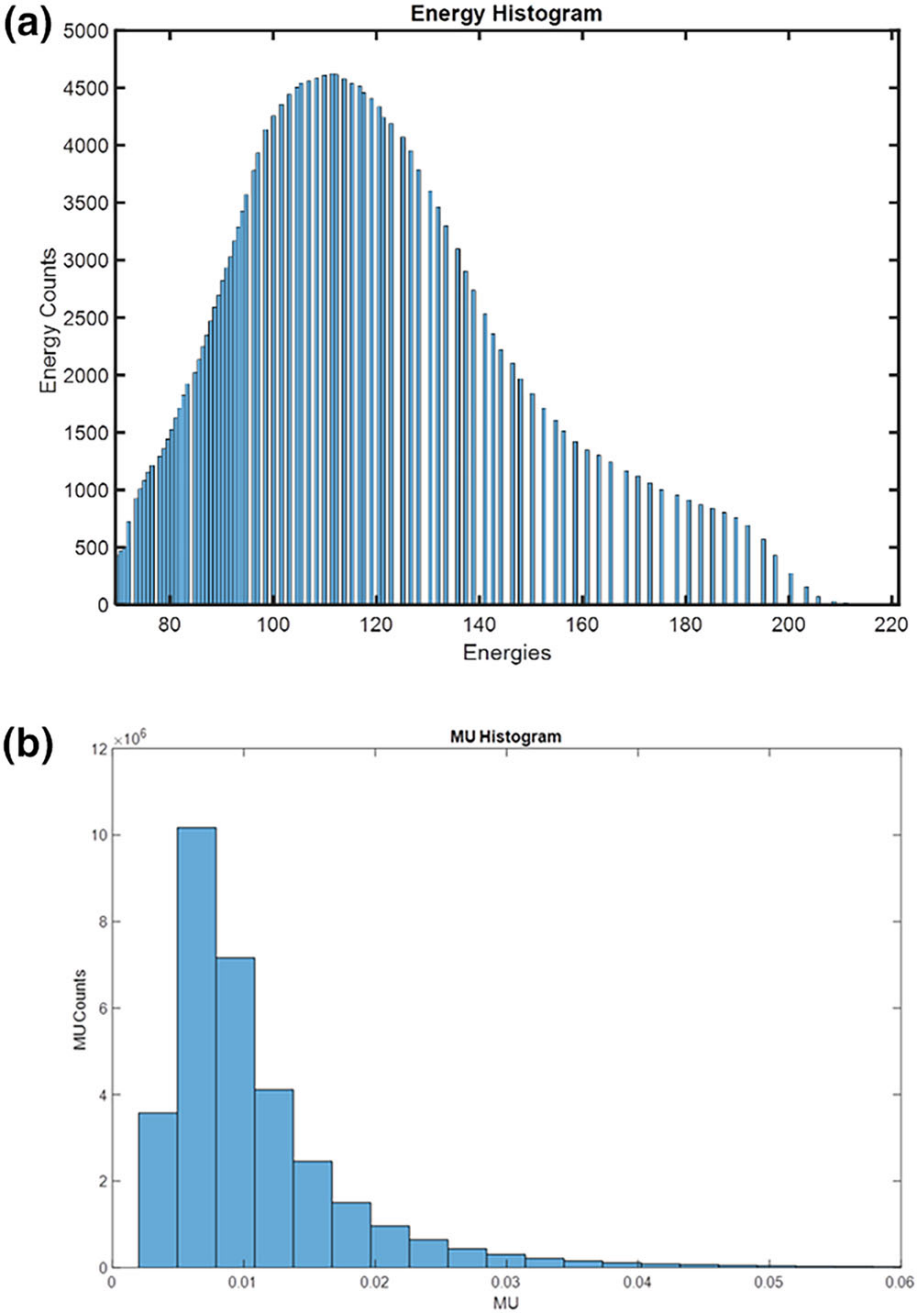
Planned energy (a) and MU (b) histograms plotted for more than 32 million spots generated by the TPS.

Over the course of 6 years from 2016 to 2021, the trend analysis showed that the proton delivery system was very stable in delivering patient QA fields with proton energies of 69.4–221.3 MeV and with 0.003–1.015 MU per spot. The planned mean and standard deviation (SD) of the energy were 114.4 ± 26.4 MeV, which would correspond to a water equivalent thickness of 9.8 cm in this range. The planned mean and SD of the spot MU were 0.010 ± 0.009 MU. The histograms corresponding to the planned energy layer and MU are shown in Figure 2. Considering the minimum deliverable MU of 0.003 MU per spot, the mean planned MU was approximately three times larger than the minimum deliverable MU set on the proton machine.

**FIGURE 3:**
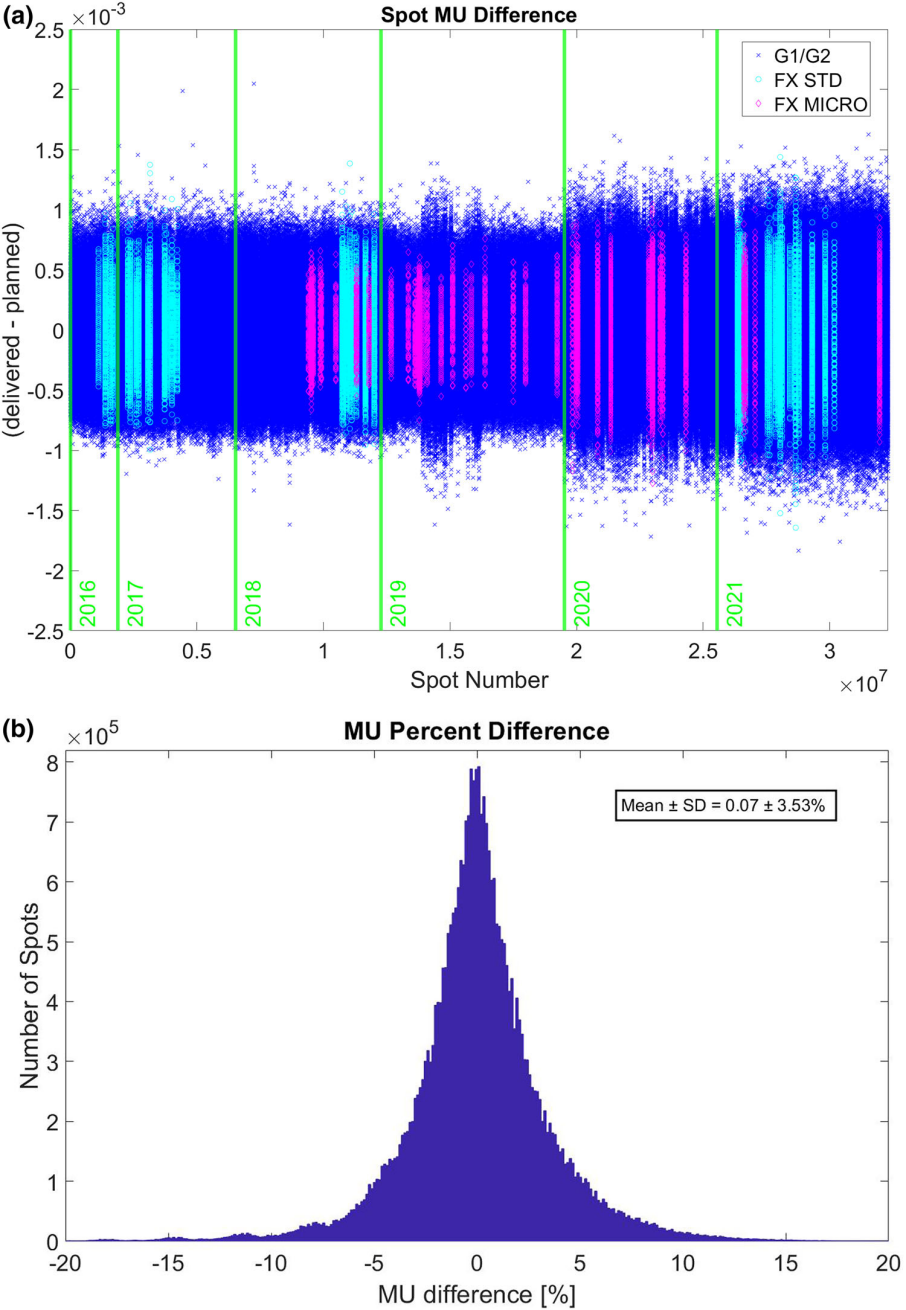
Spot MU (a), and spot MU percent (b) differences between delivered and planned spots for the 6 years from 2016 to 2021. Data for the interchangeable G1/G2 rooms and the FX room with standard (STD) beams and micro‐beams are color coded in spot MU difference (a).

In log file–based proton patient QA, the spot MU difference was found by subtracting the MU of the delivered spot from that of the planned spot for each spot generated by the TPS and recorded as delivered by the MOSAIQ R&V system. The Hitachi PROBEAT‐V system at our institution has two half‐gantry rooms (G1 and G2) and one fixed (FX) room that has standard beam and micro‐beam capabilities. Whereas the G1 and G2 rooms are interchangeable by virtue of having the same beam models, the FX room has its own micro‐beam model. Figure 3a shows that the mean and SD of the difference in the spot MU between the delivered and planned spots were 9.56 × 10^−8^ ± 2.0 × 10^−4^ MU over the 2016–2021 period. Figure 3b presents a histogram for the MU percent difference of each delivered spot compared to the planned spot to emphasize the magnitude of MU percent deviation for all gantry rooms including all the data. In the histogram, the mean and SD of the percent difference in the spot MU between delivered and planned spots were 0.07 ± 3.53%.

**FIGURE 4:**
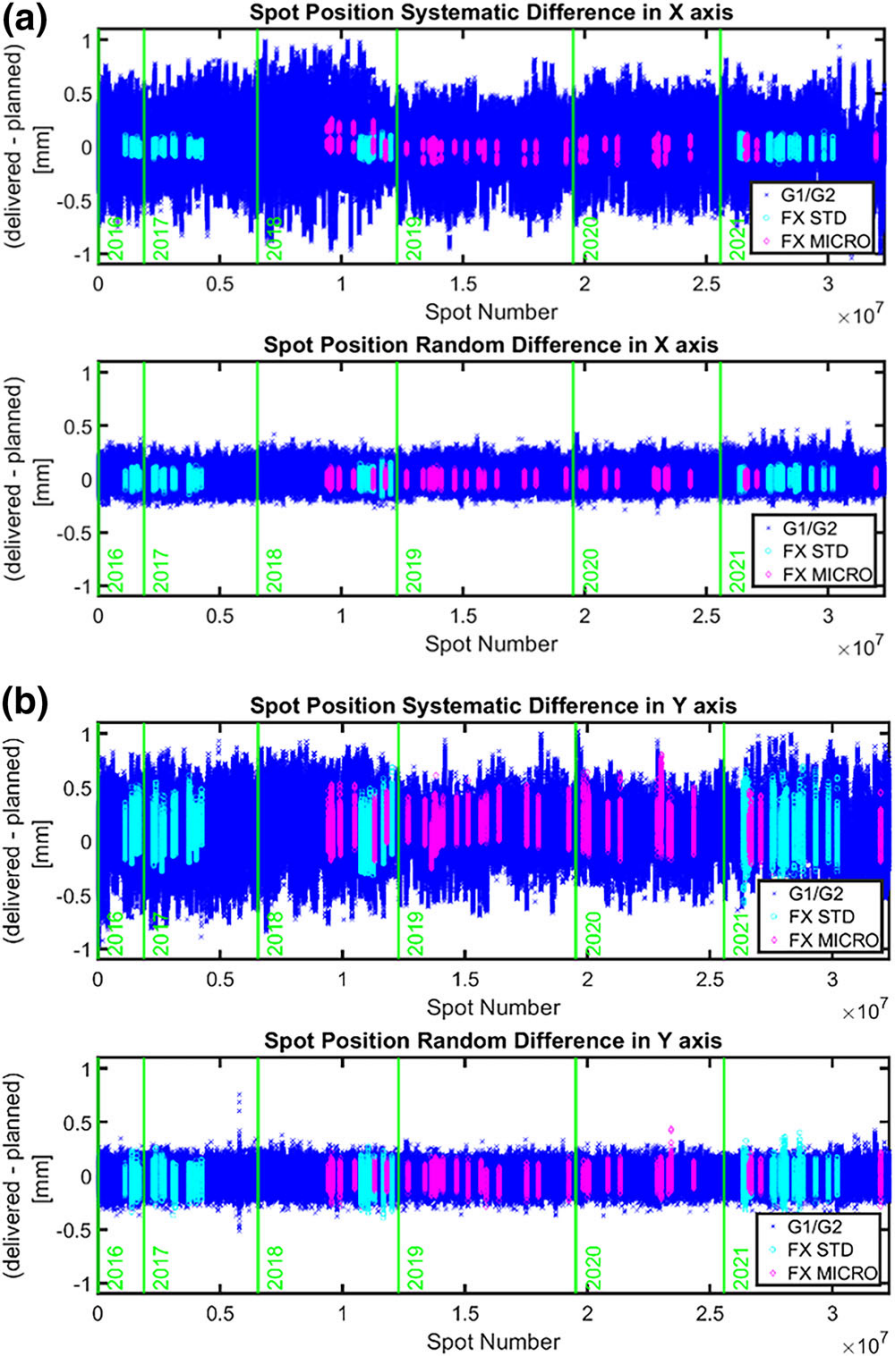
Positional differences on the X‐axis (a) and Y‐axis (b) for systematic (top) and random (bottom) errors between delivered and planned spots for the 6 years from 2016 to 2021.

The mean and SD of the positional differences between the delivered and planned spots were 0.029/−0.007 ± 0.049/0.044 mm on the X/Y‐axis for random positional differences and 0.005/0.125 ± 0.189/0.175 mm on the X/Y‐axis for systematic positional differences. Figure 4 shows the spot position differences on both the X‐ and Y‐axes, with color coding indicating the results for individual gantry rooms. Although the statistical random errors were relatively small because they resulted from individual spot displacements, the systematic errors were much larger because of the number of accumulated spots in a given energy layer. However, all positional errors were less than ±1 mm, which corresponds to a spot position tolerance of ±1 mm.

**FIGURE 5:**
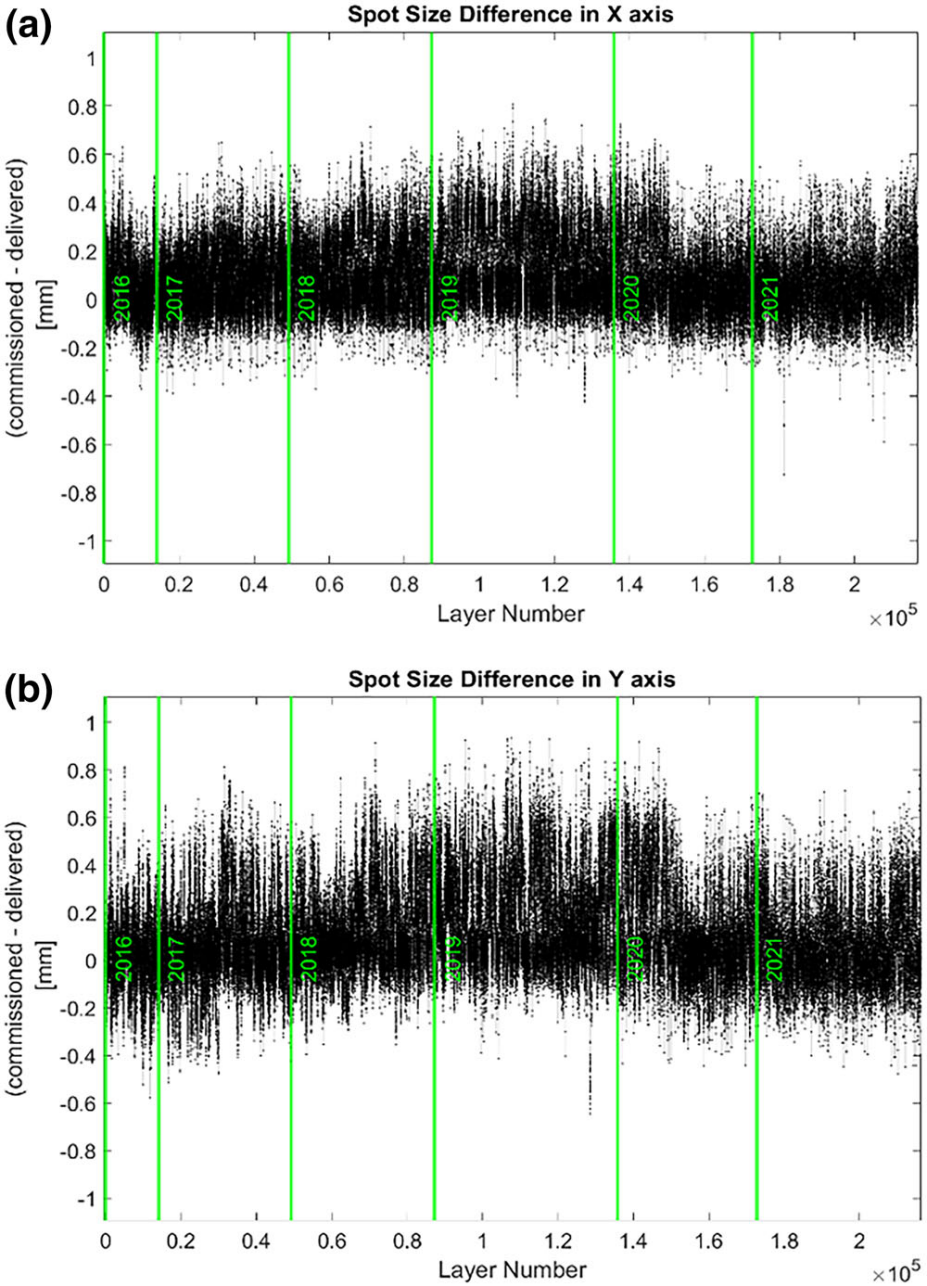
Spot size differences on the X‐axis (a) and Y‐axis (b) between commissioning and delivered spots for the 6 years from 2016 to 2021.

When the commissioning and delivered spot sizes were compared, the mean and SD of the positional differences were 0.086/0.089 ± 0.131/0.166 mm on the X/Y‐axis. Figure 5 illustrates the spot size differences for the 2016–2021 period. The errors in the size difference between the commissioning and delivered spots were less than ±1 mm for all energy layers used in the TPS. The spot size tolerance was ±10% from the baseline values.

Overall, the log file–based proton patient QA has detected no delivered spots that exceeded the machine tolerances set at the time of commissioning for spot MU, spot position, or spot size on the lateral X‐ and Y‐axes.

Ten randomly selected CSI cases were investigated for offline plan review using the delivered log‐files. As it targets one of the largest disease sites, CSI requires a large number of beam spots in the TPS to include the cranial and spine regions. Table 1 shows the details of 10 CSI cases with two target volumes (designated CTV Cranium and CTV Spine). For each case, the dose prescriptions and the dose–volume parameters, including the minimum, maximum, and mean doses (Dmin, Dmax and Dmean, respectively) are listed. The target coverages for the original plan were taken from the approved treatment plan in the TPS. Dose–volume parameters for the reconstructed plans were re‐calculated based on the delivered log files, using original CT data and structures in the TPS. Gamma analysis was performed based on a 3% dose difference and a 3‐mm distance to agreement, with a passing rate of 90% or higher during proton patient QA for the original plans quoted in the table.

**TABLE 1:** Data for 10 CSI cases with the Dmin, Dmax, and Dmean values for the original and reconstructed target volumes and the gamma passing rates of the original plans.

Patient Number	Age (Years)	Treatment Year	Rx (Gy[RBE])	Original CTV Cranium (%)	Cranium Gamma (%)	Reconstructed CTV Cranium (%)	Original CTV Spine (%)	Spine Gamma (%)	Reconstructed CTV Spine (%)
				Dmin/Dmax/Dmean		Dmin/Dmax/Dmean	Dmin/Dmax/Dmean		Dmin/Dmax/Dmean
1	10	2016	23.4	97.7/108.9/103.9	95.8	97.0/110.4/104.0	92.3/105.5/100.8	92.6	93.0/107.7/101.8
2	8	2016	15.0	96.2/114.9/105.8	95.7	96.2/119.4/106.2	99.4/110.1/105.9	94.4	99.7/111.9/106.6
3	3	2017	36.0	95.2/108.4/100.3	97.6	96.9/114.3/105.8	95.8/104.8/100.1	97.2	98.1/110.1/104.6
4	7	2017	15.0	94.7/108.8/102.8	96.4	94.5/111.1/103.4	98.5/106.4/102.5	96.8	98.4/108.0/103.1
5	4	2018	23.4	94.4/106.6/100.7	97.3	92.9/105.9/99.7	94.9/102.5/98.8	94.7	93.4/101.9/97.8
6	18	2018	15.0	97.0/109.3/104.2	98.3	97.0/111.2/104.9	99.0/107.6/103.4	91.6	97.7/114.1/104.3
7	12	2019	23.4	94.7/103.8/99.3	97.8	92.8/103.0/97.8	95.8/102.7/99.0	97.6	93.3/101.1/97.5
8	12	2019	23.4	93.3/106.7/99.1	98.6	92.9/106.6/99.1	95.2/103.0/98.9	98.8	94.0/103.3/98.8
9	15	2020	23.4	94.2/101.7/97.7	98.5	92.8/101.8/97.3	95.1/100.4/97.9	93.4	93.1/101.0/97.6
10	8	2020	23.4	93.8/106.2/98.4	98.8	93.3/105.8/98.3	95.0/101.6/98.2	97.5	94.5/102.3/98.2

**FIGURE 6:**
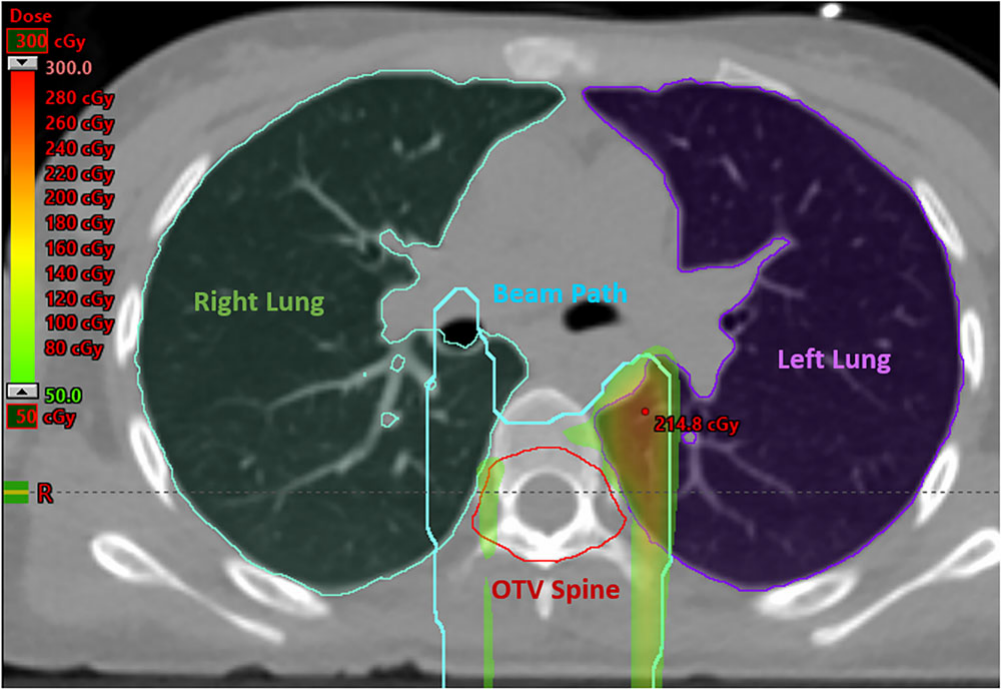
The difference in dose between the reconstructed and original plans for patient #6 indicates a hot region in the left lung that received 2.1 Gy(RBE) more than was originally planned, as shown in the transverse plane of the CSI plan. The beam path represents the 20% isodose line. OTV Spine is the expansion of CTV Spine for plan optimization.

All the CSI cases passed the target volume constraints and gamma analysis, but patient #6 stood out from the rest by having the lowest gamma passing rate of 91.6%. The same case also had the largest difference (6.5% in the Dmax of CTV Spine) between the original and reconstructed plans among the various dose–volume parameters and targets. To examine the spine region more closely, the two plans were subtracted from each other. This revealed that a major discrepancy had occurred in the left lung region, with a difference of +2.1 Gy(RBE), even though the lung constraints specified by the clinical protocol were still met. Figure 6 shows the difference in the 3D plan dose between the reconstructed and original plans in the TPS.

## 4 DISCUSSION

In our proton therapy clinic, every treatment plan undergoes a well‐established patient QA procedure, in which absolute dose measurements are made using a 2D ion‐chamber array detector (MatriXX PT, IBA Dosimetry, Schwarzenbruck, Germany). In the proton patient QA procedure, two dose planes are selected from the flat region of the SOBP in the TPS to be measured at the two corresponding depths, using a solid‐water phantom. In conjunction with the proton patient QA, log file–based proton patient QA has been performed for every proton case and has yielded a passing result, based on the standards and specifications of the machine.

Reconstructing delivered dose distributions in the TPS is not necessarily a part of the standard proton patient QA procedure. However, there is value in verifying the reconstructed dose distributions in an offline plan review before commencing the actual treatment. Verifying reconstructed dose distributions as a pre‐treatment QA provides an additional layer of safety with regard to the performance of the machine, both qualitatively and quantitatively. Qualitative analysis is performed in the TPS when the dose constraints are verified for both the target volumes and the OAR volumes, which are adjacent to the target volumes.

With regard to future directions, a comprehensive statistical analysis of the data for more than 32 million proton spots may reveal correlations in terms of spot MU, spot position, and spot size errors that can be associated with the disease site, the patient QA gamma analysis, the location and magnitude of highly weighted spots, the proton range and modulation, the beam orientations, the plan parameters and constraints, and/or the single‐field uniform dose versus multi‐field optimization methods in IMPT. If a critical organ dose is found to be adversely and consistently affected in reconstructions of dose distributions and in statistical data, changing the beam angles and plan constraints could be considered to facilitate a toxicity analysis. Statistical analysis may also reveal that poor gamma analysis for certain disease sites correlates with larger spot MU differences and highly weighted spots, especially when heterogeneities are concerned. If statistically significant results are obtained for certain energy layers, ranges, or modulations, the proton beam models can be revisited and perfected using the TPS. Although a comprehensive statistical analysis is beyond the scope of this study, that would be the next step in this QA program after having collected information on more than 32 million spots from 992 pediatric proton patients.

## 5 CONCLUSION

From 2016 to 2021, more than 32 million proton spots in 992 pediatric patients were analyzed using an in‐house log file–based proton patient QA algorithm. No spots were found that exceeded the machine tolerances with respect to spot MU, spot position, or spot size on the lateral X‐ and Y‐axes. The spot MU differences were within 10^−4^ MU, and the position and size differences between delivered and planned spots were within ±1 mm.

The spot‐based analysis that verifies the spot list of the original plan quantitatively is an integral part of the proton patient QA program. Although this is a comprehensive check at the granular level, including the spot MU, position, and size, verification that spots are within the machine specifications may not always translate qualitatively to the treatment plan. Therefore, reconstructed dose calculations based on delivered log files are valuable additions to the proton patient QA, in which the quality of the treatment plan is verified against the plan constraints, especially when there is concern regarding a critical structure adjacent to the target volumes. The reconstruction of the daily treatment delivery is also valuable for offline adaptive proton therapy and OAR toxicity analysis when the reconstructed dose is accumulated for every fraction to depict the true form of the treatment delivery.

## AUTHOR CONTRIBUTIONS

Ozgur Ates: Design of the work, analysis, drafting, final approval, Fakhriddin Pirlepesov: Interpretation of data for the work, drafting, final approval, Li Zhao: Interpretation of data for the work, drafting, final approval, Chia‐Ho Hua: Drafting the work, analysis, drafting, final approval, Thomas E. Merchant: Drafting, interpretation of data for the work, final approval.

## CONFLICT OF INTEREST STATEMENT

There is no conflict of interest for the authors.

## Data Availability

The data that support the findings of this study are available on request from the corresponding author. The data are not publicly available due to privacy or ethical restrictions.
